# Do emotional demands and exhaustion affect work engagement? The mediating role of mindfulness

**DOI:** 10.3389/fpsyg.2024.1432328

**Published:** 2024-10-14

**Authors:** Merve Karahan Kaplan, Gözde Bozkurt, Bumin Çağatay Aksu, Serdar Bozkurt, Ayşe Günsel, Gülşah Gencer Çelik

**Affiliations:** ^1^Işık University, Psychology, Istanbul, Türkiye; ^2^Istanbul Beykent University, Economics, Istanbul, Türkiye; ^3^Istanbul Gelişim University, Healthcare Management, Istanbul, Türkiye; ^4^Yildiz Technical University, Business Administration, Istanbul, Türkiye; ^5^Kocaeli University, Business Administration, Kocaeli, Türkiye; ^6^Istanbul Beykent University, Business Administration, Istanbul, Türkiye

**Keywords:** emotional demands, emotional exhaustion, mindfulness, work engagement, nursing management

## Abstract

**Aim:**

The current paper seeks to elucidate the interrelationships among emotional demands (ED), emotional exhaustion (EE), mindfulness, and work engagement (WE), with an explanation of the mediating role of mindfulness within indicated relationships.

**Background:**

Nurses working in a stress-related environment face some emotional challenges. New methods such as mindfulness should be learned; therefore, positive outcomes occur along with new developments.

**Method:**

Responses were received from nurses through a self-report questionnaire using the convenience sample technique. Four hundred and twenty-nine nurses from health institutions in Istanbul participated in the study. The PLS-SEM technique was used to test the research model.

**Results:**

ED and mindfulness, EE and mindfulness, and mindfulness and WE relationships were found. While mindfulness was a mediator between EE and WE, it was not a mediator between ED and WE.

**Conclusion:**

It has been revealed that nurses cope with job-related challenges by being present at the moment with high awareness. Furthermore, mindful nurses also foster positive outcomes.

**Implications for nursing management:**

Precautions should be taken because of the nursing shortage. It is better to provide mindfulness training to nursing students in schools before their professional lives. Implementing and using technologies might be helpful for nurses.

## 1 Introduction

The primary goal of nursing activities is to safeguard, maintain, or aid individuals in all aspects of their lives. According to the International Council of Nurses (ICN), nursing aims to retain and rehabilitate health, prevent illness, and alleviate pain. As caregivers, nurses are interested in all aspects of care to alleviate physical pain and provide holistic care (Potter and Perry, 1994; Velioğlu, 1999). Nurses face various stressors in their workplace (Botha et al., 2015) because of working in a job that is directly related to human life, and there is no room for mistakes in nursing (Kaya et al., 2010). Moreover, nurses often face undesirable experiences while caring for patients or interacting with other healthcare team members (Penque, 2019). In addition to challenges intrinsic to the profession, nurse shortages persist due to inadequate staffing in healthcare institutions and flawed workforce policies (Çetin-Aydın et al., 2021). As per 2022 OECD healthcare resources data, the number of nurses per 1,000 inhabitants varies across countries. In the top five OECD countries, Finland has 18.9, Switzerland 18.4, Norway 18.3, Ireland 15.2, and Iceland 15.1 nurses per 1,000 inhabitants. In contrast, Türkiye, an emerging country, reports 2.7 nurses per 1,000 people. The primary cause of the nurse shortage is the high turnover due to the professional challenges of nursing (Mert-Haydari et al., 2016). Accordingly, considering the current and future problems in the nursing workforce, issues related to improving the work environment of nurses have become the focus of many studies in Türkiye (Bayer and Gölbaşı, 2021; Uzun and Mayda, 2020; Yaprak and Seren, 2010).

Healthcare workers, including nurses, face intense burnout due to employee shortages and profession-related challenges (Uzun and Mayda, 2020; Köroğlu and Bahar, 2021; Jun et al., 2021). As Xanthopoulou et al. (2009) stated burnout is associated with low work engagement and turnover, leading to organizational-level consequences. Mindfulness approaches are implemented to create a healthier work environment for nurses, promoting stress coping (Penque, 2019). Mindfulness, considered the third wave of Cognitive Behavioral psychotherapy, is effective in dealing with negative situations (Vatan, 2016), especially in professions like nursing that require a high level of mindfulness (Aşık and Albayrak, 2021). Despite the perpetual stress inherent in nursing, there is a growing emphasis on teaching stress-coping strategies during nursing education. Many School of Nursing directors report trauma, stress, and burnout as leading causes for nurses leaving the profession. The COVID-19 epidemic has further increased the number of nurses leaving their jobs. Stress, a constant phenomenon in the nursing profession, remains a phenomenon that cannot be easily overcome.

**Figure 1:**
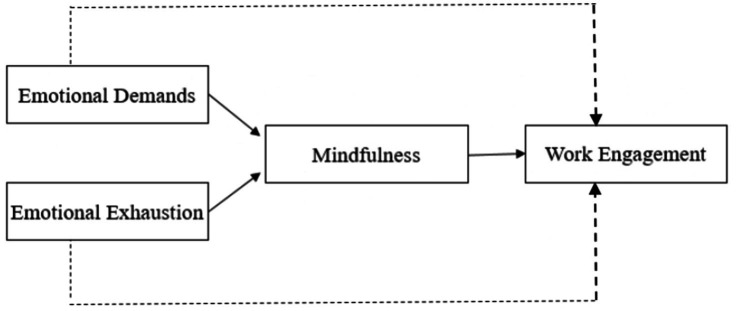
Research model. The mediation hypotheses are depicted with dashed lines.

While there is extensive research on work engagement in professions involving intensive interpersonal communication, limited studies focus on nurses’ work engagement and its outcomes (Gabel-Shemueli et al., 2014; Hosseinpour-Dalenjan et al., 2017). Mindfulness, a relatively new concept, has received limited attention in the literature, with few studies on nurses’ mindfulness levels in different countries. This study explores how nurses’ various challenges impact their work engagement. Data was obtained from nurses in Türkiye to examine the underlined problem. Additionally, the study hypothesizes the mediating effect of mindfulness between emotional exhaustion (EE), emotional demands (ED), and work engagement (WE). High mindfulness levels might enable individuals to manage environmental demands, internally balance stress, and enhance resilience (Glomb et al., 2011). Our research model is summarized in the Figure 1. The study, based on the literature, investigates (i) to unveil the relationship between ED, EE, and mindfulness among nurses in Türkiye, (ii) to identify the relationship between mindfulness and WE, and (iii) to elucidate the mediating role of mindfulness in indicated relationships.

## 2 Theoretical background and hypotheses development

### 2.1 Emotional demands and emotional exhaustion

To grasp the essence of ED and EE, it is essential to regard them as independent variables within the framework of the current study. ED, the first variable, entails coping with emotions such as anger, frustration, sadness, and helplessness in the workplace. These emotions are considered sources of emotional labor in work interactions (Totterdell and Holman, 2003; Peng et al., 2010). ED are also recognized as antecedents of emotional labor, which subsequently contributes to employee burnout (Xanthopoulou et al., 2013; Grandey, 2000; Yıldırım and Erul, 2013; Koçak and Gürsoy, 2018).

Initially conceived to comprehend burnout, and to explain WE in detail, the Job Demand-Resources (JD-R) model is discussed through a broader perspective (Schaufeli, 2017). The JD-R model, which involves job demands and job resources (Bakker and Demerouti, 2007), generates the basis for revealing the relationship between ED and WE in the current paper. Job demands reflect the requirements and conditions of the job. On the other hand, Demerouti et al. (2001) stated that job-related resources meet job demands and that these resources consist of physical, psychosocial, and organizational factors. Essentially, the JD-R model interconnects these two psychological processes, whereby high job demands and low resources induce a stress process leading to burnout. In contrast, increasing job resources fosters a motivational process resulting in positive outcomes through engagement (Schaufeli and Taris, 2014).

Consequently, the coexistence of high job demands and insufficient resources leads to destructive organizational and individual consequences (Schaufeli, 2017). Employees with low personal resources lack control over their environment, perceiving demands as obstacles (Crawford et al., 2010). Hence, their inadequate response to ED impedes WE, reinforcing the importance of personal resources in this dynamic (Sweetman and Luthans, 2010).

On the other hand, EE is the adaptive challenge arising from the depletion of emotional resources for individuals engaged in emotionally intense job roles (Maslach et al., 2001). Identified as the most common and challenging sub-dimension of burnout syndrome (Sürgevil-Dalkılıç, 2014), EE precedes cynicism and reduced personal accomplishment, key symptoms of burnout (Kristensen et al., 2005; Taris et al., 2005). Moreover, it is considered foundational to burnout, gradually reducing individual resources (Cordes and Dougherty, 1993). EE manifests as a reaction in individuals subjected to high emotional workloads (Oğuzberk and Aydın, 2008).

Underpinning these observations are job characteristics encompassing job resources and demands (Bakker et al., 2003). Workload, time pressure, and ED exemplify job demands, while social support from colleagues, self-efficacy, and autonomy represent job resources (Demerouti et al., 2001). The absence of employee recognition, extra demands, and low resources underscores an unfavorable situation for employee health (Heuven and Bakker, 2003).

In conclusion, ED and exhaustion emerge as critical issues for nursing professionals, impacting their personal and organizational outcomes (Van den Broeck et al., 2008). Understanding factors that diminish positive work outcomes and implementing remedial measures against negative conditions is imperative (Cordes and Dougherty, 1993). The empowerment of nurses in the health sector becomes crucial, given the challenging nature of their profession (Jun et al., 2021). Therefore, our study aims to elucidate strategies for addressing the adverse consequences of ED and EE, enhancing the overall well-being of nursing professionals.

### 2.2 Mindfulness

Throughout history, mindfulness has been foundational to Buddha’s discipline and has manifested in diverse ways across various religions and traditions (Brown et al., 2007). As a milestone, mindfulness has crossed its borders and has become a popular concept in the West, thanks to Dr. Jon Kabat-Zinn’s establishment of the Mindfulness-Based Stress Reduction Clinic in 1979 (Schmidt, 2011). Since there are various definitions in the literature on mindfulness, it is not easy to make a single definition. It is generally defined as a nonjudgmental awareness of one’s current experience and attention to a particular way and the present moment (Kabat-Zinn, 1994). According to Marlatt and Kristeller (1999), mindfulness focuses on the moment’s benefits while prioritizing acceptance and compassion. Siegel et al. (2009) defined mindfulness as accepting the experiences of the moment with kindness and without judgment.

According to Shapiro and Carlson (2009), the concept of mindfulness is a combination of being aware of the moment and also showing it as behavior. Moreover, Kabat-Zinn explains the concept as having a certain purpose or intention, attention, and the emergence of an attitude (Shapiro et al., 2006). Additionally, Brown et al. (2007) propose that mindfulness has both a trait-like character (a tendency to be mindful in everyday life) and a state-like character (attention to internal and external experiences).

Studies on mindfulness have increased significantly with the development of positive psychology over the last several decades (Verrier and Day, 2022). The realm of positive psychology has witnessed a surge in mindfulness studies, demonstrating its robust impact on stress coping, creativity, attention, sensitivity, self-confidence, and learning (Verrier and Day, 2022; Langer and Moldoveanu, 2000). In healthcare, mindfulness practices among nurses enhance interactions with patients, mitigate stress-related issues, boost coping abilities, elevate job satisfaction, decrease burnout levels (Mackenzie et al., 2006; Praissman, 2008), and contribute to reduced anxiety and depression, thereby improving mental health and job performance (Guillaumie et al., 2017). Mindfulness helps the individual increase his/her resources and cope with his/her job more easily. Akin to job resources in managing job demands, personal resources significantly impact personal outcomes (Schaufeli and Taris, 2014; Bakker and Demerouti, 2017).

As outlined by Bakker and Demerouti (2017), personal resources serve two key functions: buffering the destructive effects of job demands and amplifying the constructive effects. In the literature, mindfulness is posited as a personal resource with positive outcomes (Taylor and Millear, 2016). Mindful individuals excel in attention maintenance and adeptly control attentional resources, enhancing their ability to manage demands from both themselves and their environment (Good et al., 2016).

Previous studies suggest mindfulness correlates with increased WE (Janssen et al., 2020; Leroy et al., 2013) and decreased EE in nurses (Suleiman-Martos et al., 2020). When faced with negative events, mindful individuals exhibit awareness without excessive focus on the event itself, reducing self-criticism and fostering self-understanding (Atalay, 2019). In light of earlier results, we hypothesize that nurses’ ED and EE are related to their WE. Therefore, we propose the following hypotheses:

*H1: There is a negative relationship between ED and mindfulness.*

*H2: There is a negative relationship between EE and mindfulness.*

### 2.3 Work engagement

This study centers on WE as the dependent variable within positive psychology. Positive Organizational Behavior (POB), an expression of positive psychology in the organizational context, focuses on identifying and developing individual strengths rather than weaknesses (Seligman and Csikszentmihalyi, 2000). It presents a fresh approach to human resource development and management, emphasizing individuals’ strengths, optimal performance, and positive experiences in the workplace (Wright, 2003).

WE, a key topic within POB, is *“the individual’s physical, cognitive, and emotional commitment to work”* (Kahn, 1990). It has also been positioned as the antithesis of burnout (Hallberg et al., 2007; Maslach and Leiter, 1997), highlighting energy, involvement, and self-efficacy (Maslach and Leiter, 1997). In contrast to burnout, engagement signifies a more enduring, affective-cognitive state without focusing on specific objects, events, individuals, or behaviors (Schaufeli et al., 2006). Three sub-dimensions are utilized to explain WE such as vigor, absorption, and dedication. It creates positive outputs such as being more optimistic and well-being at work (Schaufeli and Bakker, 2004).

While vigor underlines being strong, patient, and flexible with high energy when faced with tough situations, dedication means that the individual is involved in his/her work, and gives a sense of challenge, and inspiration. Absorption entails an intense and joyful immersion in work, a perception of time passing swiftly, and challenges in disengaging from work (Schaufeli et al., 2002).

The literature on WE reveals its positive implications for individuals and organizations. It is inversely related to intention to quit, employee turnover, and absenteeism while positively correlating with productivity, performance, and job satisfaction (Balcı and Ağ, 2020; Caesens and Stinglhamber, 2014). Additionally, WE fosters organizational citizenship behavior (Saks, 2006) and is associated with high psychological capital, individual resource creation, improved performance, and overall happiness (Bakker et al., 2011). Studies in healthcare organizations underscore the positive effects of WE among surgeons and dentists (Mache et al., 2014).

Despite the prevalence of studies indicating varying levels of WE among nurses, it is recognized as a vital factor in the nursing profession, impacting performance and organizational outcomes (Bhatti et al., 2018; Harter et al., 2002). However, some studies highlight the challenge of low WE and high stress among nurses compared to other healthcare professionals (Fasoli, 2010; Van Bogaert et al., 2017).

Furthermore, numerous studies (e.g., Bargagliotti, 2012; Salanova et al., 2011; Fasoli, 2010; Halbesleben and Wheeler, 2008; Bhatti et al., 2018) suggest positive outcomes for nurses with high WE when facing job-related challenges, including enhanced performance, increased job interest, and overall success in the health system.

Kahn (1990) also indicates a positive relationship between psychological presence and WE, as well as a similar connection between mindfulness and WE (Langer and Moldoveanu, 2000). Thus, based on the results of previous studies, we developed the following hypothesis:

*H3: There is a positive relationship between mindfulness and WE.*

In the context of burnout studies, particularly those involving nurses, the nature of their profession, often referred to as “people work,” is considered a significant contributing factor (Maslach, 1982; Maslach and Schaufeli, 1993). Studies in the healthcare sector consistently reveal that job resources positively affect WE (e.g., Şahin et al., 2018; Başoda, 2017; Gabel-Shemueli et al., 2014). For instance, job control, self-esteem, and organizational factors are identified as significant contributors to WE (Mauno et al., 2007).

Conversely, employees facing high ED must regulate their emotions, leading to increased emotional overload (Peng et al., 2010). ED thus poses a hindrance to the development of WE (Van den Broeck et al., 2008). EE, a feeling of depletion of one’s resources, is a critical issue in the hospital sector and requires proactive measures for prevention (Woo et al., 2020; Cougot et al., 2022).

The current study anticipates that nurses facing challenges such as ED and EE may enhance their WE through mindfulness and being present in the moment. Accordingly, the following hypotheses are proposed:

*H4: Mindfulness mediates the relationship between ED and WE.*

*H5: Mindfulness mediates the relationship between EE and WE.*

## 3 Methods

### 3.1 Design and participants

In our research, data collection was conducted at public and university hospitals in Istanbul, Türkiye. Before engaging with the nurses, we obtained permission from the hospital authorities, specifically the medical superintendents (MS) and hospital directors. These authorities were fully briefed on the study’s aims, objectives, and data utilization, ensuring ethical compliance in our interactions with respondents.

To address ethical concerns, we comprehensively explained the study’s purpose and objectives through a cover letter accompanying the consent form. We informed the participants that participation in the current study was based on voluntary and that they could give up contributing to the study as participants whenever they wished.

A total of 479 nurses were sent the survey to complete; however, 442 of them completed the survey. Before moving on to the analysis phase, when the suitability of the surveys was examined for analysis, it was decided that the analysis started with only 429 surveys. The demographic profile of the participants in this convenience sample indicated that the majority, 85.5% (*n* = 367), were female. Additionally, 48% of the participants fell within the age range of 26–41 years, while 285 participants were married, comprising 66.4% of the sample. Regarding educational attainment, 78.8% (*n* = 338) held at least a bachelor’s degree. Furthermore, 132 participants (30.8%) reported having been employed within the same organization for 1–5 years.

### 3.2 Measures

A concise summary of the 5-point Likert-style measures is provided below:

- *Emotional Demands:* This composite second-order variable, comprising two sub-dimensions– emotional charge and complaints, was measured using a 6-item scale. Three questions were allocated to each sub-dimension, adapted from Van Veldhoven and Meijman (1994). Sample items include “Facing emotionally charged situations in my work” (emotional charge) and “Being confronted with people who complain continuously” (complaints).
- *Work Engagement*: Another composite second-order variable, encompassing three sub-dimensions – vigor, absorption, and dedication was measured with a 9-item scale. Three questions were assigned to each sub-dimension, adapted from Schaufeli et al. (2006). Example items include “At my work, I feel bursting with energy” (vigor), “I am proud of the work that I do” (dedication), and “I feel happy when I am working intensely” (absorption).
- *Emotional Exhaustion*: This was measured with a 7-item scale, a sub-scale of the Maslach Burnout Inventory (MBI), adapted from Maslach and Jackson (1986). Sample items include “I feel used up at the end of the workday” and “I feel burned out from work.”
- *Mindfulness*: There are four sub-dimensions: attention, awareness, present focus, and acceptance, and three expressions in each dimension. A composite second-order variable was defined with these four sub-dimensions. The relevant concept is measured with a 12-item scale adapted by Feldman et al. (2007). Examples of items include “It is easy for me to concentrate on what I am doing” (attention), “I’m able to keep track of my thoughts and feelings” (awareness), “I am able to focus on the present moment” (present focus), and “I can tolerate emotional pain” (acceptance).

### 3.3 Data analysis

To test our research model and hypotheses, we used the PLS-SEM (Least Squares Structural Equation Modeling) technique, which allows complex relationships between variables to be analyzed simultaneously. It does not seek the covariance-based structural equation modeling assumptions, which can predict (Fornell and Larcker, 1981). At the same time, PLS-SEM is far from imposing assumptions with normal distribution on error terms and indicators (Hair et al., 2014). This feature of PLS-SEM fits quite well with the nature of our data. Additionally, PLS-SEM compatibility is beneficial in analyzing sample sizes exceeding 30. Considering that our study model includes 13 latent variables consisting of first-and second-order variables, PLS-SEM seems to be a fitting technique for the research model.

## 4 Results

### 4.1 Measurement model

Reflective scales specified by Kleijnen et al. (2007) were used for the four variables used in the research. First, we estimated a null model without any relational links to reveal the psychometric properties of the scales we used for each structure. On the other hand, composite scale reliability (CR), Cronbach alpha, and average variance extracted (AVE) calculations were made for the reliability and validity of the scales. All measured PLS-based values were above the threshold value. Cronbach alpha values of acceptance and present focus both exceed 0.60, which yields an acceptable level due to their CR values exceeding 0.70.

Table 1 illustrates the correlations among the seven variables, providing additional evidence of discriminant validity. The results in our model demonstrate that none of the inter-correlations among the constructs exceed the square root of the AVE of the constructs (refer to Table 1). This finding reinforces the discriminant validity of our measures.

**Table 1:** Reliability and validity.

	1	2	3	4	5	6	7	8	9	10
Absorption	0.845									
Acceptance	0.366	0.748								
Attention	0.470	0.478	0.823							
Awareness	0.305	0.563	0.627	0.799						
Dedication	0.721	0.347	0.392	0.237	0.888					
Compliant	−0.412	−0.175	−0.271	−0.090	−0.556	0.807				
Emotional charge	−0.145	0.015	0.019	0.131	–0.244	0.574	0.822			
Exhaustion	–0.465	–0.145	–0.210	–0.063	–0.606	0.830	0.560	0.824		
Present focus	0.309	0.585	0.589	0.544	0.392	–0.276	–0.059	–0.269	0.714	
Vigor	0.681	0.342	0.422	0.269	0.639	–0.519	–0.249	–0.584	0.383	0.912
Cronbach’s alpha	0.808	0.613	0.765	0.716	0.864	0.739	0.775	0.921	0.654	0.898
CR	0.882	0.791	0.863	0.841	0.917	0.841	0.862	0.936	0.752	0.937
AVE	0.714	0.560	0.677	0.639	0.788	0.651	0.676	0.679	0.509	0.832

Diagonals show the square root of AVEs. CR, composite reliability; AVE, average variance extracted; α, Cronbach’s alpha.

Furthermore, we assessed convergent validity by scrutinizing the standardized loadings of the measures on their respective constructs. All measures exhibited standardized loadings exceeding 0.60, as detailed in the Appendix. This reaffirms the validity and reliability of our measurement model.

Moreover, since we used ED, mindfulness, and WE as composite variables, we also performed second-order factor analyses of those constructs. Second-order factor analyses are given below.

### 4.2 Emotional demands

**Figure 2:**
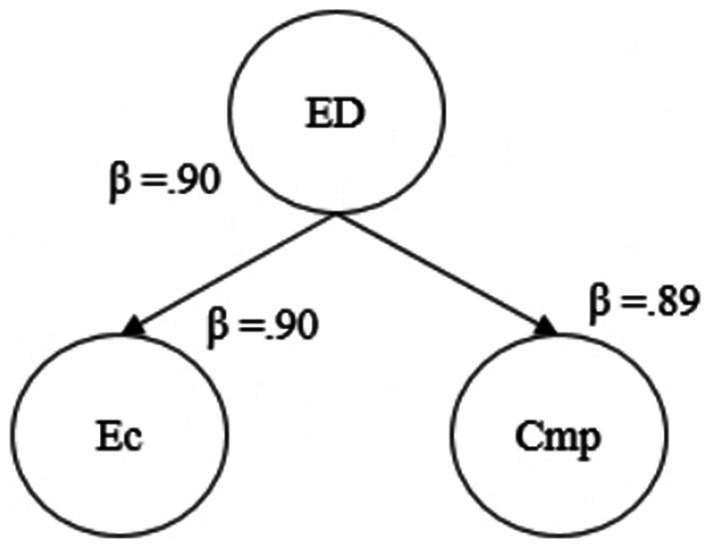
Second-order factor analysis of emotional demands.

We assumed ED as a second-order construct composed of 2 sub-dimensions- emotional charge, and complaints. Figure 2 shows the standardized regression loadings of those given two constructs as a result of second-order factor analysis. As seen in Figure 2, all two constructs exceed a standardized loading of over 0.60. This result suggests that ED as a two-dimensional second-level variable is significantly predicted by emotional charge, and complaints.

### 4.3 Mindfulness

**Figure 3:**
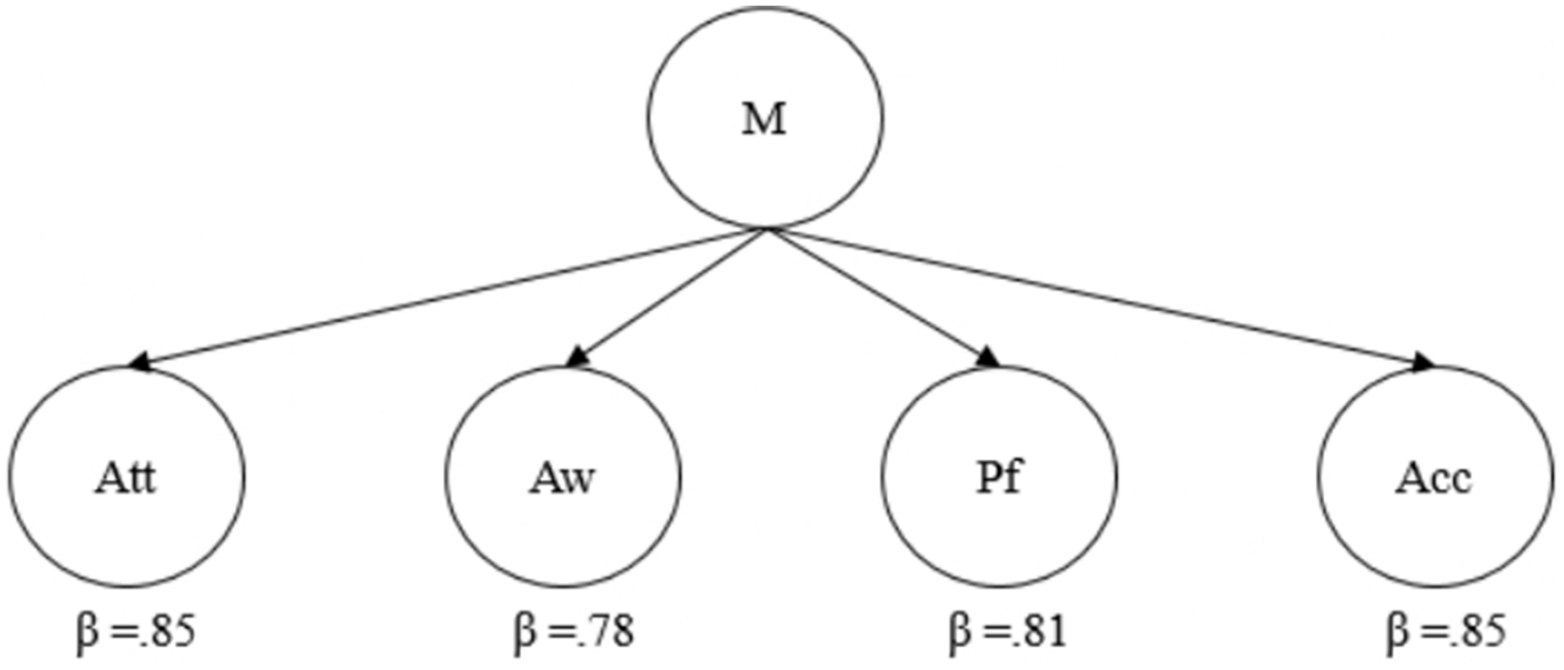
Second-order factor analysis of mindfulness.

We assumed mindfulness as a second-order construct composed of 4 sub-dimensions - attention, awareness, present focus, and acceptance. Figure 3 shows the standardized regression loadings of those given four constructs due to second-order factor analysis. As seen in Figure 3, all four constructs exceed a standardized loading of over 0.60. This result suggests that mindfulness as a four-dimensional second-level variable is significantly predicted by attention, awareness, present focus, and acceptance.

### 4.4 Work engagement

**Figure 4:**
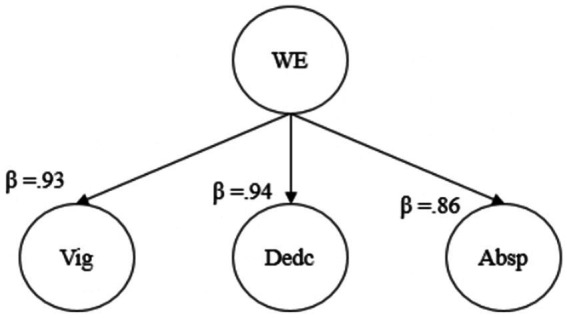
Second-order factor analysis of work engagement.

We assumed WE as a second-order construct composed of 3 sub-dimensions– vigor, dedication, and absorption. Figure 4 shows the standardized regression loadings of those given three constructs as a result of second-order factor analysis. As seen in Figure 4, all three constructs exceed a standardized loading of over 0.60. This result suggests that WE as a three-dimensional second-level variable is significantly predicted by vigor, dedication, and absorption.

### 4.5 Test of hypotheses

Our hypotheses were evaluated using the PLS (Partial Least Squares) approach (Chin et al., 2003). Hypothesis tests were assessed with Smart PLS 3.0 software, and the bootstrap resampling method was used. This method facilitated estimating main and indirect effects, hypothesis testing, and predictive power assessment for our proposed research model (see Figure 1 above).

Firstly, examining direct effects, our results indicate a negative relationship between EE and mindfulness (*β* = −0.31; *p* < 0.01), confirming a significant inverse association. Conversely, a positive and significant correlation exists between mindfulness and WE (*β* = 0.31; *p* < 0.01), supporting H2 and H3. A statistically significant positive relationship emerged between ED and mindfulness (*β* = 0.14; *p* < 0.05), leading to the non-support of Hypothesis 1 (Table 2).

**Table 2:** Path results.

Hypothesis	Relationships	*β*	Results
H1	Emotional demands → Mindfulness	0.14*	Not supported
H2	Emotional exhaustion → Mindfulness	−0.31**	Supported
H3	Mindfulness → Work engagement	0.47**	Supported

*p < 0.05, **p < 0.01.

We further conducted a mediation analysis to examine the mediating role of mindfulness in the relationship between ED, EE, and WE. Our findings reveal that EE negatively and indirectly relates to WE through mindfulness, supporting H5. However, empirical evidence for the statistically significant indirect relationship between ED and WE is not found (Table 3).

**Table 3:** Results for the mediating analyses.

Indirect effect			
Relationship	Path coefficient (*β*)	BI [2.5%;97.5%]	
ED → M → WE	0.066	−0.002	0.149
EE → M → WE	−0.148**	−0.241	−0.083

ED, emotional demands; EE, emotional exhaustion; M, mindfulness; WE, work engagement. *p < 0.05, **p < 0.01.

Within the scope of the research, we measured the coefficient of determination (R2) and the Goodness-of-Fit Index (GoF) values through PLS-SEM to evaluate model fit. While the R2 value is used to determine the amount of variance explained by endogenous variables (Barclay et al., 1995), GoF measures have been proposed as baselines to validate the PLS model (Tenenhaus et al., 2005).

The multiple regression model depends on R2, and if the R2 value is between 0.02 and 0.12, the model is weak, between 0.13 and 0.25, it is moderate, and 0.26 and above is substantial (Chin, 1998). GoF seeks harmony between the structural model and measurement performance. GoF also takes a value between 0 and 1. Accordingly, it is assumed that the path model estimation performs better as the GoF value approaches 1. The cut-off value of GoF small = 0.1, GoF medium = 0.25, and GoF large = 0.36 (Fornell and Larcker, 1981). Additionally, AVE ≥ 0.5 confirms the convergent validity.

Table 4 presents R2 and GoF values as fit measures. Mindfulness (R2 = 0.23) demonstrates a medium effect size, while WE (R2 = 0.37) indicates a large effect size. The GoF result of 0.29 signifies a medium-sized fit.

**Table 4:** Structural model.

Fit measures	Endogenous constructs	*R* ^2^
	Mindfulness	0.23
	Work engagement	0.37
GoF: 0.29		

GoF = √Average Communality × Average R2.

## 5 Discussion

In the healthcare sector, known for its labor-intensive and service-oriented nature, nurses play a pivotal role and encounter various challenges such as burnout, low job engagement, dissatisfaction, and high turnover rates (Fasoli, 2010; Jenaro et al., 2010; Xanthopoulou et al., 2009). Particularly, the Job Demands-Resources (JD-R) model, which elucidates employees’ WE, burnout, and stress levels, suggests that distinct job resources act as a buffer between job demands and WE (Bakker et al., 2007).

This study focuses on mindfulness as a concept to mitigate individual and organizational outcomes resulting from ED and EE due to job demands. Mindfulness, situated within the realm of positive psychology, has gained attention for its favorable effects in the workplace, including enhancing nurse–patient relationships, reducing stress and burnout, alleviating anxiety and depression, and fostering effective coping strategies (Guillaumie et al., 2017; Mackenzie et al., 2006). With the proliferation of positive psychology, research on mindfulness has increased (Verrier and Day, 2022).

Drawing upon existing literature, this study delved into the complex interplay among ED, EE, mindfulness, and WE within a sample of nurses in Türkiye. Initially, our findings corroborate prior research (e.g., Bekircan et al., 2023; Arslan, 2018), revealing a negative correlation between EE and mindfulness. This result shows that nurses who feel high EE are less inclined to show mindfulness in the work environment. Such a negative relationship highlights the importance of considering mindfulness in different organizational contexts, especially in professions with high levels of ED, such as nursing.

On the other hand, contrary to our hypotheses, we revealed a positive relationship between ED and mindfulness and a negative relationship between EE and mindfulness. This result highlights that these concepts have complex dynamics for nursing. Considering that mindfulness is regarded as an individual resource, it is a coping mechanism nurses use to meet workplace demands, especially in reducing EE.

Additionally, we examined the impact of mindfulness on WE. Our findings are consistent with previous research (e.g., Erol-Korkmaz, 2023; Janssen et al., 2020; Kaçay et al., 2020; Karacaoğlan and Hisli-Şahin, 2016; Leroy et al., 2013), and a positive relationship exists between mindfulness and WE. Mindfulness and engagement to work are based on positive psychology; they share common characteristics such as focus on work and concentration with dedication. Individuals with high levels of WE are known for developing their resources and exhibiting high levels of psychological capital (Bakker et al., 2011).

Finally, our study examined the mediating effect of mindfulness in the relationships between ED, EE, and WE. Our findings show that mindfulness mediates the relationship between EE and WE and that there is an indirect relationship between these variables. On the other hand, our research results do not provide empirical support for the mediating role of mindfulness in the relationship between ED and WE. This result shows that there is no indirect relationship between these variables. These findings are consistent with the existing literature indicating a negative relationship between WE and EE (Cougot et al., 2022; Başoda, 2017). EE, the central element of burnout, remains a significant concern in nursing. On the other hand, nurses working in high-stress environments can be encouraged to use mindfulness as a coping mechanism, thus increasing their WE.

In conclusion, the current study explains the relationships between ED, EE, WE, and mindfulness factors in nursing. The findings indicate the importance of mindfulness as a valuable resource for managing the challenging conditions and ED of the nursing profession and creating a high level of WE.

### 5.1 Limitations

While our study provides valuable results, it is essential to note that it has limitations that can guide future research. The fact that the research sample consists only of nurses limits the generalizability of the results to other healthcare professionals or employees in different sectors. Future studies could similarly examine labor-intensive occupations. Again, cross-cultural comparisons involving samples from various countries may reveal significant relationship differences between variables. The use of scales is a limitation in quantitatively measuring mindfulness. Future research could introduce an experimental design to evaluate the effect before and after the intervention by providing mindfulness training to a different sample. This approach may provide a clearer understanding of the relationship between mindfulness and the variables in question. Additionally, the study investigates the positive aspects of organizational behavior and emphasizes that mindfulness is a resource for nurses.

Future research could screen the broader spectrum of relationships involving mindfulness, extending beyond burnout to encompass various dimensions of nurses’ experiences and WE. This expansion could provide a more comprehensive understanding of the multifaceted nature of nurses’ well-being and performance.

In addressing these limitations, future research can build upon the current study’s foundation, contributing to a more comprehensive and nuanced understanding of the relationships between ED, EE, WE, and mindfulness across diverse professional contexts and cultural settings.

### 5.2 Practical implications

The findings of this study offer practical insights for addressing challenges within the nursing profession and enhancing nurses’ well-being. Several recommendations and implications emerge as follows.

Given the documented low WE and high EE among nurses, addressing nursing shortages is crucial. Practical measures, such as increasing the number of nurses per patient, should be considered. Studies have shown that a higher patient-to-nurse ratio is associated with increased patient deaths and nurse burnout (Saraee et al., 2017). Investing in new employment opportunities within the healthcare sector could help alleviate these issues. Introducing mindfulness training for nursing students could be a proactive approach to equip future nurses with stress-coping mechanisms early in their careers. Incorporating mindfulness into nursing education can contribute to the development of resilient and emotionally equipped professionals. This training could potentially lead to a more sustained and fulfilling career journey for nurses. With the growing investments in machine learning and AI technologies in healthcare, their integration can be explored to support nurses in their roles. AI technologies can assist nurses in providing more efficient and personalized patient care, potentially mitigating some of the stressors associated with the profession. Implementing such technologies may enhance nurses’ capabilities and contribute to improved patient outcomes. Researchers must examine the relationship between mindfulness and WE in labor-intensive healthcare sectors. In particular, addressing variables that arise from the impact of job demands and resources and contribute to employee well-being may contribute to identifying gaps in the existing literature and an in-depth understanding of their effects. These practical applications for nurses aim to improve their well-being and contribute to the overall development of the nursing profession. In summary, healthcare sector managers can increase patient care effectiveness by identifying organizational-level activities and training for developing nurses and improving their engagement in their work.

## 6 Conclusion

Our study contributes significantly to explaining the relationships between ED, EE, mindfulness, and WE for nurses and our in-depth understanding of the relationships between concepts. There are some critical findings from our research.

Our research reveals a positive relationship between ED on nurses and mindfulness. This consequence of mindfulness suggests that nurses may use it as a coping mechanism if they encounter emotional difficulties in the workplace. At the same time, mindfulness serves as a resource for navigating and managing the ED of nursing. Research shows a negative relationship between EE and mindfulness. This indicates that nurses’ mindfulness levels will decrease as they experience EE. It also highlights the potential role of mindfulness in mitigating the negative impact of facing intense ED on nurses by acting as a buffer against EE. Our research shows a positive relationship between mindfulness and WE. This finding indicates that nurses who improve mindfulness are more likely to increase their engagement to work. In this context, mindfulness is essential in enhancing nurses’ engagement to work and overall positive work experience. Our research underscores the role of mindfulness in transforming negative states, such as ED and EE, into positive outcomes, such as WE. Mindfulness serves as a mechanism through which nurses can cope with the challenges of their profession, improve their psychological resilience, and positively impact their overall work experience. The study makes significant contributions to the developing literature on mindfulness and WE within the framework of the nursing profession. By revealing the interplay between ED, EE, mindfulness, and WE, our research provides valuable information that can inform both academic discourse and practical interventions in the healthcare sector. This study advances our understanding of the psychological processes in the nursing profession and highlights the potential benefits of incorporating mindfulness practices to support nurses in their challenging roles. It opens avenues for further research and underscores the importance of holistic approaches to enhance the well-being and engagement of healthcare professionals.

## Data Availability

The original contributions presented in the study are included in the article/Supplementary material, further inquiries can be directed to the corresponding author.
