# Supplementary material for: Do emotional demands and exhaustion affect work engagement? The mediating role of mindfulness
Source: Front Psychol. 2024 Oct 14;15:1432328. doi: 10.3389/fpsyg.2024.1432328 (PMC11513345; doi:10.3389/fpsyg.2024.1432328)
Supplement: Supplementary file 1 [file Table_1.docx]

**APPENDIX**

Factor loadings are shown in the parentheses.

**Emotional Demands**: adapted from Van Veldhoven and Meijman (1994)

1. Emotional Charge of Job

Items measuring emotional charge:

- My work is emotionally demanding. (0.87)
- In my work, I am confronted with things that personally touch me. (0.78)
- I face emotionally charged situations in my work. (0.80)

2. Dealing with Users’ Complaints

Items measuring complaints:

- In my work, I deal with patients who incessantly complain, although I always do everything to help them. (0.90)
- In my work, I have to deal with demanding patients. (0.93)
- My work require that I convince/persuade people. (0.52)

**Work Engagement**: adapted from Schaufeli, Bakker, and Salanova (2006)

1. Vigor

Items measuring vigor:

- At my work, I feel bursting with energy. (0.92)
- At my job, I feel strong and vigorous. (0.93)
- When I get up in the morning, I feel like going to work. (0.87)

2. Absorption

Items measuring absorption:

- I feel happy when I am working intensely. (0.83)
- I am immersed in my work. (0.84)
- I get carried away when I am working. (0.86)

3. Dedication

Items measuring dedication:

- I am enthusiastic about my job. (0.94)
- My job inspires me. (0.93)
- I am proud of the work that I do. (0.78)

**Emotional Exhaustion**: adapted from Maslach and Jackson (1986)

Items measuring emotional exhaustion:

- I feel emotionally drained from my work. (0.78)
- I feel used up at the end of the workday. (0.76)
- I feel fatigued when I get up in the morning and have to face another day on the job. (0.82)
- Working with people all day is really a strain for me. (0.85)
- I feel burned out from my work. (0.91)
- I feel frustrated by my job. (0.87)
- I feel I’m working too hard on my job. (0.73)

**Mindfulness**: adapted from Feldman et al. (2007)

1. Attention

Items measuring attention:

- It is easy for me to concentrate on what I am doing. (0.85)
- I am not easily distracted. (0.82)
- I am able to pay close attention to one thing for a long period of time. (0.78)

2. Awareness

Items measuring awareness:

- I can usually describe how I feel at the moment in considerable detail. (0.73)
- It’s easy for me to keep track of my thoughts and feelings. (0.86)
- I try to notice my thoughts without judging them. (0.80)

3. Present Focus

Items measuring present focus:

- I am not preoccupied by the future. (0.66)
- I am preoccupied by the past. (0.59)
- I am able to focus on the present moment. (0.85)

4. Acceptance

Items measuring acceptance:

- I can tolerate emotional pain. (0.82)
- I can accept things I cannot change. (0.77)
- I am able to accept the thoughts and feelings I have. (0.63)
